# Carbapenem-resistant Enterobacterales/Enterobacteriaceae (CRE) in Indonesia: protocol for systematic review and meta-analysis

**DOI:** 10.12688/f1000research.157380.1

**Published:** 2024-10-17

**Authors:** Ika N. Kadariswantiningsih, Maulana A. Empitu, Bulat Idrisov, Derren David Christian Homenta Rampengan, Roy Novri Ramadhan

**Affiliations:** 1 Department of Medical Microbiology, Faculty of Medicine, Airlangga University, Surabaya, East Java, Indonesia; 2 Clinical Microbiology Residency Program, Dr. Soetomo Regional Hospital/ Faculty of Medicine, Airlangga University, Surabaya, East Java, Indonesia; 3 Faculty of Medicine, Airlangga University, Surabaya, East Java, Indonesia; 4 Faculty of Health, Medicine and Natural Sciences (FIKKIA), Airlangga University, Banyuwangi, East Java, Indonesia; 5 Health Services Research Department of Health Systems and Population Health, University of Washington, Seattle, Washington, USA; 6 Faculty of Medicine, Universitas Sam Ratulangi, Manado, North Sulawesi, Indonesia

**Keywords:** Carbapenem-resistant Enterobacterales, CRE, antimicrobial resistance, prevalence, Indonesia, infectious disease

## Abstract

**Background:**

Carbapenem-resistant Enterobacterales (CRE) pose a significant global health threat, with increasing prevalence worldwide, including in Indonesia. Despite the public health impact, comprehensive data on the burden of CRE in Indonesia remains fragmented. This protocol outlines a systematic review and meta-analysis aiming to estimate the prevalence of CRE in Indonesia, summarize trends over time, and identify key resistance mechanisms.

**Methods:**

We will conduct a systematic search across multiple electronic databases, including PubMed, Scopus, and local Indonesian databases, for studies reporting the prevalence of CRE in Indonesia from 2008 to 2024. Eligibility criteria include observational studies (cross-sectional, cohort, and case-control) and surveillance reports. Data extraction will focus on CRE prevalence, bacterial species, sample types, resistance mechanisms, and study settings (hospital vs. community). Quality assessment of studies will be performed using the Newcastle-Ottawa Scale (NOS). Meta-analysis will be conducted using a random-effects model to estimate pooled prevalence, and subgroup analysis will explore variations by geographical region, period, and healthcare setting.

**Discussion:**

This systematic review and meta-analysis will provide the first comprehensive overview of CRE prevalence in Indonesia, contributing to an improved understanding of the national burden and resistance patterns. The findings will guide public health policies and inform antimicrobial stewardship efforts in Indonesia.

**Registration:**

PROSPERO CRD42024580177

## Introduction

The emergence of carbapenem-resistant Enterobacterales (CRE) is a global health challenge, with Southeast Asia experiencing particularly high prevalence rates of these multidrug-resistant organisms. The widespread dissemination of carbapenemase-producing Enterobacteriaceae (CPE) has been well documented, driven by both clonal expansion and horizontal gene transfer. ^1, 2^ Within Southeast Asia, including Indonesia, carbapenem resistance rates are among the highest globally, with some healthcare settings reporting resistance rates exceeding 30%. ^3, 4^ This trend underscores the urgency of implementing targeted interventions to curtail the spread of CRE.

Carbapenem resistance in Enterobacterales is mediated through complex mechanisms. The primary mechanism is the production of various carbapenemases such as *Klebsiella pneumoniae* carbapenemase (KPC), New Delhi metallo-β-lactamase (NDM), and oxacillinase-48 (OXA-48). ^5, 6^ These enzymes degrade carbapenems, rendering them ineffective against infections once treatable with these last-resort antibiotics. Furthermore, resistance is compounded by additional factors such as efflux pumps and modifications to porin channels, further complicating therapeutic options. ^7, 8^ A comprehensive understanding of these mechanisms is essential to developing effective surveillance and intervention strategies for CRE control in Indonesia.

In Indonesia, studies suggest that the prevalence of CRE varies across different regions. The variation is influenced by factors like antibiotic usage patterns, healthcare practices, and the presence of specific resistance genes. ^9, 10^ This systematic review and meta-analysis will critically assess the prevalence and mechanisms of CRE resistance in Indonesia, contributing to the global effort to combat this escalating health crisis. The findings aim to inform healthcare policy and improve the management of CRE infections both within Indonesia and on a broader scale.

## Protocol

### Review question

This review seeks to answer the following question: What is the prevalence, antimicrobial resistance patterns, and clinical outcomes of Carbapenem-resistant Enterobacterales/Enterobacteriaceae (CRE) infections in Indonesia?

### Study design and protocol registration

This systematic review and meta-analysis are designed to estimate the pooled prevalence of Carbapenem-resistant Enterobacterales (CRE) in Indonesia from 2004 to 2024, following the Preferred Reporting Items for Systematic Reviews and Meta-Analyses Protocols (PRISMA-P) guidelines. ^11^ The PRISMA-P Checklist is detailed in the Extended data. The review will systematically identify and analyze published studies to provide a comprehensive assessment of CRE prevalence, trends, and resistance mechanisms across different settings in Indonesia. The review protocol has been registered in the International Prospective Register of Systematic Reviews (PROSPERO) under registration number CRD42024580177.

### Data source and search strategy

An exhaustive database search was conducted to gather all pertinent articles on Carbapenem-resistant Enterobacterales (CRE) in Indonesia, covering the period from January 1, 2004, to the start of this study. Publications in both English and Indonesian were sought in databases including PubMed, Google Scholar, Scopus, and Southeast Asian Index Medicus between January 1, 2004 and the start of this study, 2024. Additionally, a manual search using Google was performed to screen the references of identified articles, ensuring comprehensiveness and the inclusion of potentially missed studies.

The search strategy was developed using the CoCoPop (Condition, Context, Population) framework to structure the search questions and terms. The search terms included: (“Carbapenem-resistant Enterobacterales”), (CRE), (“prevalence”), and (“Indonesia”). These terms were combined using Boolean operators “OR” and “AND” to optimize and refine the search, ensuring the retrieval of all relevant articles. The search strategy is detailed in Appendix 1 (Extended data), and the flowchart of this systematic review is depicted in Appendix 2 (Extended data).

### Eligibility criteria

This review includes studies involving patients or clinical isolates from all age groups (neonates, children, adults) that have been tested for Carbapenem-resistant Enterobacterales (CRE) in both community-acquired and hospital-acquired (nosocomial) infections. Only studies conducted in Indonesia will be included to ensure accurate representation of local epidemiology and CRE burden. The eligible study designs include observational studies (cohort, cross-sectional, case-control) and clinical trials that report on the prevalence, resistance patterns, clinical outcomes, or any measure of disease burden related to CRE. Both published and grey literature (e.g., theses, dissertations, conference proceedings) in English and Indonesian will be considered. Studies must report specific outcomes, such as prevalence rates, antimicrobial resistance profiles, morbidity, mortality, or length of hospital stay associated with CRE infections.

Exclusion criteria include studies conducted outside of Indonesia or those that do not provide relevant data to the Indonesian context. Review articles, meta-analyses, systematic reviews, editorials, and opinion pieces are excluded. Additionally, studies focusing on non-human subjects, including veterinary medicine, agriculture (e.g., livestock, aquaculture), or environmental sampling (e.g., water, soil) are excluded unless directly related to human health outcomes. Studies that fail to report specific data on CRE are also excluded, as well as case reports and case series due to potential bias and challenges in generalizing findings from individual cases or small samples.

### Data extraction

A structured data extraction approach will be employed for this systematic review and meta-analysis. Initially, two authors will independently screen studies based on their titles and abstracts. Subsequently, full-text articles will be thoroughly reviewed using the predetermined inclusion and exclusion criteria to identify relevant data. A predefined data extraction sheet will be used to systematically record all relevant details from selected studies. Key information to be extracted includes authors, publication year, title, study name, the year the study was conducted, study site (city, province, region), study design, study setting (hospital, community), population (age group), sampling site, comorbidities, CRE detection method, total sample/total isolates, CRE prevalence, upper-lower confidence intervals (CI), CRE prevalence from specific pathogens (e.g., *Escherichia coli* and *Klebsiella pneumoniae*), and clinical outcomes other than prevalence (e.g., mortality, survival). Data extraction will be conducted by two authors, and a third author will cross-verify the retrieved data during the analysis phase to ensure accuracy and consistency.

### Quality assessment

The quality of the included studies will be assessed by two authors using a modified version of the Newcastle-Ottawa Scale (NOS) for observational studies. Studies receiving 0-3 stars will be considered poor quality, those with 4-5 stars acceptable, and those with 6-9 stars high quality. In cases of disagreement between the reviewers, consensus will be reached through discussion.

### Outcome variables

The primary outcome of this systematic review and meta-analysis is to determine the pooled prevalence of CRE in Indonesia. Secondary outcomes include the pooled prevalence of CRE by region, the prevalence in adults versus children, and the prevalence in hospital versus community settings. The review will also examine the prevalence of the most common CRE pathogens.

### Statistical analysis

A random effects model will be applied to estimate the pooled prevalence and antimicrobial resistance patterns of CRE. Subgroup and meta-regression analyses will be conducted to explore potential sources of heterogeneity. Heterogeneity between studies will be quantified using Cochran’s Q test and I ^2^ statistics, with a p-value of <0.05 indicating significant heterogeneity. The Der Simonian-Laird random effects model will account for this variability. Meta-Essentials, a tool compatible with Microsoft Excel, will be used for analysis. Subgroup analyses will focus on region, age group, hospital versus community settings, and specific pathogens. Results will be presented in tabular form and forest plots, and publication bias will be assessed using funnel plot symmetry.

### Dissemination

Upon completion, the systematic review will be sent for publication in a peer-reviewed journal.

### Study status

This study has not been started yet.

## Discussion

This systematic review and meta-analysis aim to provide a comprehensive overview of Carbapenem-resistant Enterobacterales (CRE) in Indonesia. The findings are expected to shed light on the growing prevalence of CRE, particularly in hospital settings, which mirrors global trends in antimicrobial resistance. This study’s results will be pivotal for shaping infection control policies and antimicrobial stewardship programs within Indonesia’s healthcare system, highlighting the need for enhanced surveillance and targeted interventions.

Regional variations in CRE prevalence are likely to emerge, influenced by disparities in healthcare infrastructure, antimicrobial usage, and infection control practices. These variations may underscore the need for localized strategies in regions with a higher burden of CRE. Additionally, the study will assess the prevalence across different population groups and between hospital and community settings, which could reveal the spread of CRE beyond healthcare facilities, complicating containment efforts and necessitating broader public health interventions.

While this review offers critical insights, it may face challenges such as heterogeneity in study designs, diagnostic methods, and the potential for publication bias. Subgroup and meta-regression analyses will be employed to address these limitations, but caution is warranted when interpreting the pooled data. Nevertheless, this analysis will provide crucial information for guiding national policies on antimicrobial resistance and informing future research and interventions to mitigate the threat posed by CRE in Indonesia.

## Ethical considerations

This systematic review is based on the analysis of published articles (secondary data) and does not require approval by an ethics committee.

## Data Availability

No data are associated with this article. Figshare: Extended dataset for: “Carbapenem-resistant Enterobacterales/Enterobacteriaceae (CRE) in Indonesia: protocol for systematic review and meta-analysis.”, https://doi.org/10.6084/m9.figshare.27134661.v5. ^12^ This project contains the following extended data:
1. Appendix 1. Search strategy for Pubmed 2. Appendix 2. PRISMA Flowchart 3. PRISMA-P checklist Data are available under the terms of the Creative Commons Attribution 4.0 International license (CC-BY 4.0). Figshare: PRISMA-P checklist for ‘Carbapenem-resistant Enterobacterales/Enterobacteriaceae (CRE) in Indonesia: protocol for systematic review and meta-analysis’, https://doi.org/10.6084/m9.figshare.27134661.v5. ^12^ Data are available under the terms of the Creative Commons Attribution 4.0 International license (CC-BY 4.0).
