# Carbapenem-resistant Enterobacterales (CRE) in Indonesia: protocol for systematic review and meta-analysis

**DOI:** 10.12688/f1000research.157380.2

**Published:** 2025-03-10

**Authors:** Ika N. Kadariswantiningsih, Maulana A. Empitu, Bulat Idrisov, Derren David Christian Homenta Rampengan, Roy Novri Ramadhan

**Affiliations:** 1 Clinical Microbiology Residency Program, Dr. Soetomo Regional Hospital/ Faculty of Medicine, Airlangga University, Surabaya, East Java, Indonesia; 2 Department of Medical Microbiology, Faculty of Medicine, Airlangga University, Surabaya, East Java, Indonesia; 3 Faculty of Health, Medicine and Natural Sciences (FIKKIA), Airlangga University, Banyuwangi, East Java, Indonesia; 4 Faculty of Medicine, Airlangga University, Surabaya, East Java, Indonesia; 5 Health Services Research Department of Health Systems and Population Health, University of Washington, Seattle, Washington, USA; 6 Faculty of Medicine, Universitas Sam Ratulangi, Manado, North Sulawesi, Indonesia

**Keywords:** Carbapenem-resistant Enterobacterales, CRE, antimicrobial resistance, prevalence, Indonesia, infectious disease

## Abstract

**Background:**

Carbapenem-resistant Enterobacterales (CRE) pose a significant global health threat, with increasing prevalence worldwide, including in Indonesia. Despite the public health impact, comprehensive data on the burden of CRE in Indonesia remains fragmented. This protocol outlines a systematic review and meta-analysis aiming to estimate the prevalence of CRE in Indonesia, summarize trends over time, and identify key resistance mechanisms.

**Methods:**

We will conduct a systematic search across multiple electronic databases, including PubMed, Scopus, and local Indonesian databases, for studies reporting the prevalence of CRE in Indonesia from 2004 to 2024. Eligibility criteria include observational studies (cross-sectional, cohort, and case-control) and surveillance reports. Data extraction will focus on CRE prevalence, bacterial species, sample types, resistance mechanisms, and study settings (hospital vs. community). Quality assessment of studies will be performed using the Newcastle-Ottawa Scale (NOS). Meta-analysis will be conducted using a random-effects model to estimate pooled prevalence, and subgroup analysis will explore variations by geographical region, period, and healthcare setting.

**Discussion:**

This systematic review and meta-analysis will provide the first comprehensive overview of CRE prevalence in Indonesia, contributing to an improved understanding of the national burden and resistance patterns. The findings will guide public health policies and inform antimicrobial stewardship efforts in Indonesia.

**Registration:**

PROSPERO CRD42024580177

## Introduction

Carbapenems are among the most critical classes of β-lactam antibiotics, often considered the **last line of defense** against multidrug-resistant (MDR) Gram-negative bacterial infections. These broad-spectrum antibiotics, including **imipenem, meropenem, doripenem, and ertapenem**, exhibit potent activity against a wide range of bacterial pathogens. ^1, 2^ Carbapenems are also reserved for treating **severe, life-threatening infections** such as **hospital-acquired pneumonia, complicated urinary tract infections, and infections in immunocompromised patients**. ^1, 2^ The efficacy of carbapenems stems from their ability to **resist hydrolysis by most β-lactamases**, including extended-spectrum β-lactamases (ESBLs), making them the preferred treatment option when first-line antibiotics fail. ^1, 2^

However, the emergence of carbapenem-resistant Enterobacterales (CRE) has severely compromised the effectiveness of these life-saving antibiotics. Southeast Asia experiences particularly high prevalence rates of CRE. The widespread dissemination of carbapenemase-producing Enterobacteriaceae (CPE) has been well documented, driven by both clonal expansion and horizontal gene transfer. ^3, 4^ Within Southeast Asia, including Indonesia, carbapenem resistance rates are among the highest globally, with some healthcare settings reporting resistance rates exceeding 30%. ^5, 6^ The presence of CRE in a patient **often indicates treatment failure**, as these bacteria exhibit resistance to nearly all β-lactam antibiotics, including penicillins, cephalosporins, and carbapenems. ^7^ Consequently, **infection with CRE is associated with higher morbidity and mortality rates**, as alternative treatment options are limited. ^7^ This trend underscores the urgency of implementing targeted interventions to curtail the spread of CRE.

Carbapenem resistance in Enterobacterales is mediated through complex mechanisms. The primary mechanism is the production of various carbapenemases such as *Klebsiella pneumoniae* carbapenemase (KPC), New Delhi metallo-β-lactamase (NDM), and oxacillinase-48 (OXA-48). ^8, 9^ These enzymes degrade carbapenems, rendering them ineffective against infections once treatable with these last-resort antibiotics. Furthermore, resistance is compounded by additional factors such as efflux pumps and modifications to porin channels, further complicating therapeutic options. ^10, 11^ A comprehensive understanding of these mechanisms is essential to developing effective surveillance and intervention strategies for CRE control in Indonesia.

In Indonesia, studies suggest that the prevalence of CRE varies across different regions. The variation of prevalence is influenced by factors like antibiotic usage patterns, healthcare practices, and the presence of specific resistance genes. ^12, 13^ A research has identified a significant presence of the New Delhi metallo-β-lactamase-1 (NDM-1) enzyme among carbapenem-resistant Gram-negative bacilli in Indonesia, suggesting its widespread dissemination. In a study focusing on urinary tract infections, 81.8% of carbapenemase-producing isolates were NDM-1 producers, predominantly found in Enterobacteriaceae and Acinetobacter species. ^14^ Comparative data from Asian countries reveal that Indonesia has one of the highest imipenem resistance rates among Enterobacterales, estimated at 5.8%, with varieties across its different regions. ^15^ This rate surpasses those reported in neighboring countries such as Vietnam (3.0%) and the Philippines (3.7%), underscoring the critical need for targeted antimicrobial stewardship and infection control measures within Indonesian healthcare settings. ^15^

This systematic review and meta-analysis will critically assess the prevalence and mechanisms of CRE resistance in Indonesia, contributing to the global effort to combat this escalating health crisis. The findings aim to inform healthcare policy and improve the management of CRE infections both within Indonesia and on a broader scale.

## Protocol

### Review question

This review seeks to answer the following question: What is the prevalence, antimicrobial resistance patterns, and clinical outcomes of CRE infections in Indonesia?

### Study design and protocol registration

This systematic review and meta-analysis are designed to estimate the pooled prevalence of CRE in Indonesia from 2004 to 2024, following the Preferred Reporting Items for Systematic Reviews and Meta-Analyses Protocols (PRISMA-P) guidelines. ^16^ The PRISMA-P Checklist is detailed in the Extended data. The review will systematically identify and analyze published studies to provide a comprehensive assessment of CRE prevalence, trends, and resistance mechanisms across different settings in Indonesia. The review protocol has been registered in the International Prospective Register of Systematic Reviews (PROSPERO) under registration number CRD42024580177.

### Data source and search strategy

An exhaustive database search was conducted to gather all pertinent articles on CRE in Indonesia, covering the period from January 1, 2004, to the start of this study. Publications in both English and Indonesian were sought in databases including PubMed, Google Scholar, Scopus, and Southeast Asian Index Medicus between January 1, 2004 and the start of this study, 2024. Additionally, a manual search using Google was performed to screen the references of identified articles, ensuring comprehensiveness and the inclusion of potentially missed studies.

The search strategy was developed using the CoCoPop (Condition, Context, Population) framework to structure the search questions and terms. The search terms included: (“Carbapenem-resistant Enterobacterales”), (CRE), (“prevalence”), and (“Indonesia”). These terms were combined using Boolean operators “OR” and “AND” to optimize and refine the search, ensuring the retrieval of all relevant articles. The search strategy is detailed in Appendix 1 (Extended data), and the flowchart of this systematic review is depicted in Appendix 2 (Extended data).

### Eligibility criteria

This review includes studies involving patients or clinical isolates from all age groups (neonates, children, adults) that have been tested for CRE in both community-acquired and hospital-acquired (nosocomial) infections. Only studies conducted in Indonesia will be included to ensure accurate representation of local epidemiology and CRE burden. The eligible study designs include observational studies (cohort, cross-sectional, case-control) and clinical trials that report on the prevalence, resistance patterns, clinical outcomes, or any measure of disease burden related to CRE. Both published and grey literature (e.g., theses, dissertations, conference proceedings) in English and Indonesian will be considered. Studies must report specific outcomes, such as prevalence rates, antimicrobial resistance profiles, morbidity, mortality, or length of hospital stay associated with CRE infections.

Exclusion criteria include studies conducted outside of Indonesia or those that do not provide relevant data to the Indonesian context. Review articles, meta-analyses, systematic reviews, editorials, and opinion pieces are excluded. Additionally, studies focusing on non-human subjects, including veterinary medicine, agriculture (e.g., livestock, aquaculture), or environmental sampling (e.g., water, soil) are excluded unless directly related to human health outcomes. Studies that fail to report specific data on CRE are also excluded, as well as case reports and case series due to potential bias and challenges in generalizing findings from individual cases or small samples.

### Data extraction

A structured data extraction approach will be employed for this systematic review and meta-analysis. Initially, two authors will independently screen studies based on their titles and abstracts. Subsequently, full-text articles will be thoroughly reviewed using the predetermined inclusion and exclusion criteria to identify relevant data. A predefined data extraction sheet will be used to systematically record all relevant details from selected studies. Key information to be extracted includes authors, publication year, title, study name, the year the study was conducted, study site (city, province, region), study design, study setting (hospital, community), population (age group), sampling site, comorbidities, CRE detection method, total sample/total isolates, CRE prevalence, upper-lower confidence intervals (CI), CRE prevalence from specific pathogens (e.g., *Escherichia coli* and *Klebsiella pneumoniae*), and clinical outcomes other than prevalence (e.g., mortality, survival). Data extraction will be conducted by two authors, and a third author will cross-verify the retrieved data during the analysis phase to ensure accuracy and consistency.

### Quality assessment

The quality of the included studies will be assessed by two authors using a modified version of the Newcastle-Ottawa Scale (NOS) for observational studies. ^17^ Studies receiving 0-3 stars will be considered poor quality, those with 4-5 stars acceptable, and those with 6-9 stars high quality. In cases of disagreement between the reviewers, consensus will be reached through discussion.

### Outcome variables

The primary outcome of this systematic review and meta-analysis is to determine the pooled prevalence of CRE in Indonesia. Secondary outcomes include the pooled prevalence of CRE by region, the prevalence in adults versus children, and the prevalence in hospital versus community settings. The review will also examine the prevalence of the most common CRE pathogens.

### Statistical analysis

A random effects model will be applied to estimate the pooled prevalence and antimicrobial resistance patterns of CRE. Prevalence and resistance rates will be extracted as proportions and transformed using logit transformation to stabilize variance. The pooled estimates will be calculated using **inverse variance weighting**, incorporating both the within-study variance and between-study variance (τ ^2^ **)**, which will be estimated using the DerSimonian-Laird method. Subgroup and meta-regression analyses will be conducted to explore potential sources of heterogeneity. Heterogeneity between studies will be quantified using Cochran’s Q test and I ^2^ statistics, with a p-value of <0.05 indicating significant heterogeneity. The Der Simonian-Laird random effects model will account for this variability. Meta-Essentials, a tool compatible with Microsoft Excel, will be used for analysis. ^18^ Subgroup analyses will focus on region, age group, hospital versus community settings, and specific pathogens. Results will be presented in tabular form and forest plots, and publication bias will be assessed using funnel plot symmetry.

### Dissemination

Upon completion, the systematic review will be sent for publication in a peer-reviewed journal.

### Study status

This study has not been started yet.

## Discussion

This systematic review and meta-analysis aim to provide a comprehensive overview of CRE in Indonesia, a nation confronting a significant public health challenge due to the high prevalence of antimicrobial-resistant organisms. ^14, 19^ The findings are expected to shed light on the growing prevalence of CRE, particularly in hospital settings, which mirrors global trends in antimicrobial resistance. This study’s results will be pivotal for shaping infection control policies and antimicrobial stewardship programs within Indonesia’s healthcare system, highlighting the need for enhanced surveillance and targeted interventions.

One of the most concerning carbapenem resistance mechanisms in Indonesia is **NDM-1**, an enzyme that hydrolyzes nearly all β-lactam antibiotics, including carbapenems, rendering them ineffective. First identified in *Klebsiella pneumoniae* and *Escherichia coli* isolates from a patient in Sweden in 2008, NDM-1 has since spread globally, with a high prevalence in South and Southeast Asia, including Indonesia. ^20, 21^ A study focusing on urinary tract infections identified 116 carbapenem-resistant Gram-negative bacilli among 1,082 isolates, with *Klebsiella pneumoniae* and *Providencia rettgeri* among the notable pathogens. ^12^ The dominant resistance mechanism identified was the production of NDM-1, accounting for 81.8% of carbapenemase-producing isolates. ^14^ Another study analyzing bloodstream infections in Indonesian intensive care units found that 31% of patients harbored CRE, with NDM-1 being the most commonly detected carbapenemase gene. ^22^ The dissemination of NDM-1-producing CRE in **nosocomial infections** raises serious concerns about the effectiveness of existing antimicrobial therapies, as these strains are often resistant to multiple antibiotic classes, including aminoglycosides and fluoroquinolones. ^22^

The spread of NDM-1 is particularly concerning because of its association with mobile genetic elements such as plasmids and transposons, which facilitate horizontal gene transfer between bacterial species. ^23^ This means that once an NDM-1-producing strain enters a healthcare facility, it can rapidly disseminate within the hospital environment, infecting immunocompromised patients and those undergoing invasive procedures such as mechanical ventilation or catheterization. ^23, 24^ Several hospital outbreaks of NDM-1-producing CRE have been reported in Indonesia, particularly in neonatal and intensive care units, where infection control measures may be less effective due to resource constraints. ^12, 22^

Regional variations in CRE prevalence are likely to emerge, influenced by disparities in healthcare infrastructure, antimicrobial usage, and infection control practices. Urban tertiary-care hospitals often report higher burdens of these resistant organisms compared to smaller healthcare facilities, suggesting that resource availability and patient demographics play critical roles in their dissemination. For instance, a study evaluating the Xpert Carba-R assay in an Indonesian intensive care unit found a 31% colonization rate of carbapenem-resistant organisms among patients, with the *NDM* gene being the most frequently detected carbapenemase gene. ^25^ These variations may underscore the need for localized strategies in regions with a higher burden of CRE. Additionally, this study will assess the prevalence across different population groups and between hospital and community settings, which could reveal the spread of CRE beyond healthcare facilities, complicating containment efforts and necessitating broader public health interventions.

While this review offers critical insights, it may face challenges such as heterogeneity in study designs, diagnostic methods, and the potential for publication bias. Subgroup and meta-regression analyses will be employed to address these limitations, but caution is warranted when interpreting the pooled data. Nevertheless, this analysis will provide crucial information for guiding national policies on antimicrobial resistance and informing future research and interventions to mitigate the threat posed by CRE in Indonesia.

## Ethical considerations

This systematic review is based on the analysis of published articles (secondary data) and does not require approval by an ethics committee. The analysis and interpretation will be conducted independently by the authors, ensuring that no external influence affects the findings.

## Author contribution statement

The roles and responsibilities of the contributors in this study will be as follows: **INK** will conceptualize the study, design the methodology, supervise the research process, and contribute to data analysis, interpretation, and manuscript drafting; **MAE** and **RNR** will participate in data collection, statistical analysis, and interpretation of findings; **BI** will provide expertise in systematic review methodology and meta-analysis, ensuring adherence to best practices; **DDCHR** will assist in literature screening, data extraction, and manuscript writing. All authors will review and approve the final manuscript and contribute to the dissemination of results.

## Data Availability

No data are associated with this article. Figshare: Extended dataset for: “Carbapenem-resistant Enterobacterales/Enterobacteriaceae (CRE) in Indonesia: protocol for systematic review and meta-analysis.”, https://doi.org/10.6084/m9.figshare.27134661.v6. ^26^ This project contains the following extended data:
1. Appendix 1. Search strategy for Pubmed 2. Appendix 2. PRISMA Flowchart 3. PRISMA-P checklist Data are available under the terms of the Creative Commons Attribution 4.0 International license (CC-BY 4.0). Figshare: PRISMA-P checklist for ‘Carbapenem-resistant Enterobacterales/Enterobacteriaceae (CRE) in Indonesia: protocol for systematic review and meta-analysis’, https://doi.org/10.6084/m9.figshare.27134661.v6. ^26^ Data are available under the terms of the Creative Commons Attribution 4.0 International license (CC-BY 4.0).
