# Supplementary material for: Carbapenem-resistant Enterobacterales (CRE) in Indonesia: protocol for systematic review and meta-analysis
Source: F1000Res. 2025 Mar 10;13:1244. Originally published 2024 Oct 17. [Version 2] doi: 10.12688/f1000research.157380.2 (PMC11979575; doi:10.12688/f1000research.157380.2)
Supplement: Peer review history

# Reviewer report: Reviewer response for version 2

**DOI:** 10.5256/f1000research.178768.r370283

**Published:** 2025-04-08

**Authors:** Oznur Caliskan-Aydogan

**Affiliations:** 1 Michigan State University, East Lansing, Michigan, USA

no further comments

Is the study design appropriate for the research question?

Yes

Is the rationale for, and objectives of, the study clearly described?

Yes

Are sufficient details of the methods provided to allow replication by others?

Yes

Are the datasets clearly presented in a useable and accessible format?

Partly

Reviewer Expertise:

Microbiology and nanotechnology

I confirm that I have read this submission and believe that I have an appropriate level of expertise to confirm that it is of an acceptable scientific standard.

# Reviewer report: Reviewer response for version 2

**DOI:** 10.5256/f1000research.178768.r370284

**Published:** 2025-03-21

**Authors:** Harvey Morgan Scott, Ingrid Maribel Leon Moreno

**Affiliations:** 1 Department of Veterinary Pathobiology, Texas A&M University (Ringgold ID: 14736), College Station, Texas, USA; 2 Department of Veterinary Pathobiology, College of Veterinary Medicine and Biomedical Sciences, Texas A&M University, College Station, TX, USA

General Comments:

 The authors have successfully integrated most of the suggested changes, improving the clarity and structure of the research review protocol.

 Specific Comments:

1. Clarification of XDR in PubMed Search Strategy:
  The abbreviation XDR in the search strategy for PubMed table should be explicitly defined. Specifying it as "extensively drug-resistant (XDR)" in the search query or providing a clear definition in the methods section would enhance clarity and ensure consistency in search results.
2. Inconsistency in Search Strategy Table and Protocol Text:
  The search strategy table includes only PubMed, whereas the protocol text under Data Source and Search Strategy mentions additional databases such as Google Scholar, Scopus, and Southeast Asian Index Medicus. The authors should ensure consistency by either updating the table to include all mentioned databases or revising the protocol text to reflect only the sources used.

Is the study design appropriate for the research question?

Yes

Is the rationale for, and objectives of, the study clearly described?

Yes

Are sufficient details of the methods provided to allow replication by others?

Partly

Are the datasets clearly presented in a useable and accessible format?

Not applicable

Reviewer Expertise:

Epidemiology, Antimicrobial Resistance Research, Foodborne Pathogens, Microbiology

We confirm that we have read this submission and believe that we have an appropriate level of expertise to confirm that it is of an acceptable scientific standard.

# Reviewer report: Reviewer response for version 1

**DOI:** 10.5256/f1000research.172818.r365246

**Published:** 2025-02-26

**Authors:** Harvey Morgan Scott, Ingrid Maribel Leon Moreno

**Affiliations:** 1 Department of Veterinary Pathobiology, Texas A&M University (Ringgold ID: 14736), College Station, Texas, USA; 2 Department of Veterinary Pathobiology, College of Veterinary Medicine and Biomedical Sciences, Texas A&M University, College Station, TX, USA

The manuscript by Kadariswantiningsih and co-authors represents a protocol for the systematic review and meta-analysis of Carbapenem-resistant Enterobacterales/ Enterobacteriaceae (CRE) regarding the prevalence of CRE in Indonesia and via various subgroups and characteristics. The protocol offers valuable insights, though it requires some minor improvements as outlined in the specific examples below.

 Comments and edits:

 Introduction

1. Explain why the treatment with the carbapenem family is so very important for human health. It likely seems self-evident to many, but if the cited prevalence data of ~30% are correct, does finding a CRE in a patient indicate treatment failure, or other outcomes (surveillance for quarantine of patients, etc).
2. Please indicate briefly that carbapenemase enzymes also break down other ‘lower-order'beta-lactam antibiotics such as third and fourth generation cephalosporins so impact is on almost all beta-lactams used for Enterobacterales and not just carbapenems.
3. Should the NOS scale have a cited reference?

Data source and search strategy

1. The search coverage in the body of the manuscript was from January 1, 2004 to 2024. Note that the abstract says only from 2008. There is an inconsistency. Was something discovered regarding the available papers from the first 4 years to render them less useful and therefore excluded? Some genes had yet to be discovered/characterized (e.g., NDM-1 in 2008). Is this the reason? If so please explain.
2. Abbreviations should be explained when mentioned for the first time, for example, MDR and XDR
3. Smaller Indonesian islands (e.g., Nussa Tenggara Islands and Maluku Islands) were not included in the search for geographical locations in Indonesia. Exclusion criteria should likewise be mentioned and likely impacts on search (i.e., will search under ‘Indonesia’ capture these?).
4. What is the impact of not including the specific search strategy of antimicrobial resistance via the carbapenemase genes, since that is included in the scope of interest. For instance: NDM, KPC, etc. Will the other search terms ensure papers focused on the genetic elements be captured?

Statistical analysis

1. Please explain how the random effects model will be used to estimate the pooled prevalence and the antimicrobial resistance patterns of CRE?
2. Is the Meta-Essentials tool, the Excel compatible software used to do the analysis capable of conducting the random effects model above? A quick looks at the software website at Erasmus University does not make this clear.

Funding statement

 Please check if the below are required for published protocols. These are common for publishing results, but it is not clear to me if these aspects of disclosure are needed at this stage.

1. It is important to explain who the sponsors and funders of the study are.
2. In the protocol, it should be clear the involvement and potential influence of any party.
3. In the protocol, it should outline the roles and responsibilities of the contributors in study design, data analysis and interpretation, writing, and dissemination of results.

Is the study design appropriate for the research question?

Yes

Is the rationale for, and objectives of, the study clearly described?

Yes

Are sufficient details of the methods provided to allow replication by others?

Partly

Are the datasets clearly presented in a useable and accessible format?

Not applicable

Reviewer Expertise:

Epidemiology, Antimicrobial Resistance Research, Foodborne Pathogens, Microbiology

We confirm that we have read this submission and believe that we have an appropriate level of expertise to confirm that it is of an acceptable scientific standard, however we have significant reservations, as outlined above.

# Response

**Published:** 2025-02-27

**Authors:** IKA NINDYA

**The manuscript by Kadariswantiningsih and co-authors represents a protocol for the systematic review and meta-analysis of Carbapenem-resistant Enterobacterales/ Enterobacteriaceae (CRE) regarding the prevalence of CRE in Indonesia and via various subgroups and characteristics. The protocol offers valuable insights, though it requires some minor improvements as outlined in the specific examples below.**

 Response: Thank you for your encouraging and positive comments. Your suggestion helps us to improved the protocol and manuscript.

**Comments and edits:**

**INTRODUCTION**

**1. Explain why the treatment with the carbapenem family is so very important for human health. It likely seems self-evident to many, but if the cited prevalence data of ~30% are correct, does finding a CRE in a patient indicate treatment failure, or other outcomes (surveillance for quarantine of patients, etc).** 

 Response: We have revised the introduction and add the explanation about this in paragraph 1.

**2. Please indicate briefly that carbapenemase enzymes also break down other ‘lower-order'beta-lactam antibiotics such as third and fourth generation cephalosporins so impact is on almost all beta-lactams used for Enterobacterales and not just carbapenems.**

 Response: We have revised the introduction and add the explanation about this in paragraph 2.

**3. Should the NOS scale have a cited reference?**

 Response: We have added a reference about NOS.

**DATA SOURCE AND SEARCH STRATEGY**

**1. The search coverage in the body of the manuscript was from January 1, 2004 to 2024. Note that the abstract says only from 2008. There is an inconsistency. Was something discovered regarding the available papers from the first 4 years to render them less useful and therefore excluded? Some genes had yet to be discovered/characterized (e.g., NDM-1 in 2008). Is this the reason? If so please explain.**

 Response: We apologize for the mistake. We have changed the abstract text to 2004 instead of 2008. We want to capture the trends within the last 20 years.

**2. Abbreviations should be explained when mentioned for the first time, for example, MDR and XDR**

 Response: In the revised manuscript, we have explained those abbreviations when when mentioned for the first time.

**3. Smaller Indonesian islands (e.g., Nussa Tenggara Islands and Maluku Islands) were not included in the search for geographical locations in Indonesia. Exclusion criteria should likewise be mentioned and likely impacts on search (i.e., will search under ‘Indonesia’ capture these?).**

 Response: We have revised the search strategy to include other smaller islands such as Bali,  Nusa Tenggara, Timor and Maluku. The exclusion criteria are mentioned in the “Eligibility Criteria” section and will be used during the screening of title, abstract and full-text.

**4. What is the impact of not including the specific search strategy of antimicrobial resistance via the carbapenemase genes, since that is included in the scope of interest. For instance: NDM, KPC, etc. Will the other search terms ensure papers focused on the genetic elements be captured?**Response: Thank you for the suggestion. Wee include the following keywords to the new search strategy:

 ("Carbapenem resistance"[MeSH] OR "Antimicrobial resistance"[MeSH] OR "Multidrug-resistant organisms"[MeSH] OR "MDR"[Title/Abstract] OR "XDR"[Title/Abstract] OR "Carbapenemase" OR "Carbapenem resistance" OR "Carbapenem-resistant" OR "Beta-lactamase" OR "β-lactamase" OR ("bla_KPC" OR "bla_NDM" OR "bla_OXA-48" OR "bla_OXA-181" OR "bla_OXA-232" OR "bla_OXA-58" OR "bla_OXA-244" OR "bla_IMP" OR "bla_VIM" OR "bla_GES" OR "bla_SME" OR "bla_KHM" OR "bla_DIM" OR "bla_AIM" OR "bla_FIM" OR "bla_SPM" OR "bla_BIC" OR "bla_IMI" OR "bla_CGB" OR "bla_CCR")

**STATISTICAL ANALYSIS**

**1. Please explain how the random effects model will be used to estimate the pooled prevalence and the antimicrobial resistance patterns of CRE?**

 Response: We have added these explanation in the revised manuscript, *“Statistical Analysis” section:*

*“A random effects model will be applied to estimate the pooled prevalence and antimicrobial resistance patterns of CRE. Prevalence and resistance rates will be extracted as proportions and transformed using logit transformation to stabilize variance. The pooled estimates will be calculated using inverse variance weighting, incorporating both the within-study variance and between-study variance (τ ^2^), which will be estimated using the DerSimonian-Laird method.”*

**2. Is the Meta-Essentials tool, the Excel compatible software used to do the analysis capable of conducting the random effects model above? A quick looks at the software website at Erasmus University does not make this clear.**

 Response: Thank you for your concern. The tool is capable of conducting the random effect model as validated here: Suurmond, R., van Rhee, H., & Hak, T. (2017). Introduction, comparison, and validation of Meta‐Essentials: a free and simple tool for meta‐analysis. *Research synthesis methods*, *8*(4), 537-553.

**FUNDING STATEMENT**

**Please check if the below are required for published protocols. These are common for publishing results, but it is not clear to me if these aspects of disclosure are needed at this stage.**

**1. It is important to explain who the sponsors and funders of the study are.**

 Response:  In the Grant information section and Funding Section, we mentioned “The author(s) declared that no grants were involved in supporting this work.”  (https://f1000research.com/articles/13-1244/v1)

**2. In the protocol, it should be clear the involvement and potential influence of any party.**Response: We have added this sentence to the “Ethical consideration” statement: “The analysis and interpretation will be conducted independently by the authors, ensuring that no external influence affects the findings.” In the “Competing interests”, we write: “No competing interests were disclosed.”

**3. In the protocol, it should outline the roles and responsibilities of the contributors in study design, data analysis and interpretation, writing, and dissemination of results.**

 Response: We added the following section:

*Author Contribution Statement:*

*The roles and responsibilities of the contributors in this study will be as follows: INK will conceptualize the study, design the methodology, supervise the research process, and contribute to data analysis, interpretation, and manuscript drafting; MAE and RNR will participate in data collection, statistical analysis, and interpretation of findings; BI will provide expertise in systematic review methodology and meta-analysis, ensuring adherence to best practices; DDCHR will assist in literature screening, data extraction, and manuscript writing. All authors will review and approve the final manuscript and contribute to the dissemination of results.*

# Reviewer report: Reviewer response for version 1

**DOI:** 10.5256/f1000research.172818.r354977

**Published:** 2025-01-28

**Authors:** Oznur Caliskan-Aydogan

**Affiliations:** 1 Michigan State University, East Lansing, Michigan, USA

General Comments

 Methods and protocol of this manuscript is detailly documented well. However, Introduction and discussion should be extended. Conclusion and future perspectives should be included. The details and data about the mentioned reviewed papers for this manuscript have not been summarized. This manuscript need to be improved and enriched with the more literature resources and their findings.  

 Specific comments

 Carbapenem-resistant Enterobacterales have been used by CDC since 2022, please update all Enterobacteriaceae to Enterobacterales.

 CRE abbrevetion is already defined in the introduction. In each section CRE is defined several times. 

 In the abstract, it is mentioned resources used from 2008 to 2024. However, it is mentioned in protocol as 2004 to 2024. Please correct it.

Is the study design appropriate for the research question?

Yes

Is the rationale for, and objectives of, the study clearly described?

Yes

Are sufficient details of the methods provided to allow replication by others?

Yes

Are the datasets clearly presented in a useable and accessible format?

Partly

Reviewer Expertise:

Microbiology and nanotechnology

I confirm that I have read this submission and believe that I have an appropriate level of expertise to confirm that it is of an acceptable scientific standard, however I have significant reservations, as outlined above.

# Response

**Published:** 2025-02-27

**Authors:** IKA NINDYA

**General Comments**

**Methods and protocol of this manuscript is detailly documented well. However, Introduction and discussion should be extended. Conclusion and future perspectives should be included. The details and data about the papers mentioned and reviewed for this manuscript have not been summarized. This manuscript need to be improved and enriched with the more literature resources and their findings.**

 Response: Thank you for your encouraging and positive comments. We add some elaboration to the previous findings and literature.

 We have revised the introduction section and added an explanation about why carbapenems are important antibiotics and what the impacts of CRE are. We also add the previous finding about the significant presence of the New Delhi metallo-β-lactamase-1 (NDM-1) enzyme in Indonesia and how the prevalence rate of imipenem resistance differs from that of other countries in the same regions.

 We have expanded the discussion to highlight the significant role of NDM-1 as a dominant carbapenem resistance mechanism in Indonesia, emphasizing its widespread prevalence in both urinary tract and bloodstream infections. We further elaborated on the impact of mobile genetic elements in facilitating the rapid dissemination of NDM-1-producing CRE within hospital environments, particularly affecting immunocompromised patients and those undergoing invasive procedures, with documented outbreaks in neonatal and intensive care units. Additionally, we addressed regional variations in CRE prevalence, linking higher burdens to differences in healthcare infrastructure, antimicrobial stewardship, and infection control practices while also underscoring the potential for CRE spread beyond hospital settings, necessitating broader public health interventions.

**Specific comments**

**1. Carbapenem-resistant Enterobacterales have been used by CDC since 2022, please update all Enterobacteriaceae to Enterobacterales.**

 Response: We have updated all Enterobacteriaceae to Enterobacterales.

**2. CRE abbrevetion is already defined in the introduction. In each section CRE is defined several times.**

 Response: Thank you. We have corrected all the redundant CRE descriptions.

**3. In the abstract, it is mentioned resources used from 2008 to 2024. However, it is mentioned in protocol as 2004 to 2024. Please correct it.**

 Response: Thank you. The text in the abstract has been revised to 2004 to 2024 instead of 2008 to 2024.
